# Kegel Exercise Training Program among Women with Urinary Incontinence

**DOI:** 10.3390/healthcare10122359

**Published:** 2022-11-24

**Authors:** Ahmad H. Abu Raddaha, Elsayeda H. Nasr

**Affiliations:** 1 Department of Nursing, College of Applied Medical Sciences, Prince Sattam Bin Abdulaziz University, Al-Kharj 11942, Saudi Arabia; 2 Department of Maternity, Obstetric and Gynecological Nursing, Faculty of Nursing, Port Said University, Port Said 42526, Egypt

**Keywords:** Kegel exercise, quality of life, urinary incontinence, training program, women

## Abstract

A common condition with a large global prevalence and a persistent medical taboo for many people is urinary incontinence. Around one in three women globally are impacted by it. The most frequently suggested physical therapy treatment for women with stress incontinence or urge incontinence is Kegel exercise (also called pelvic floor muscle training). This study aims to assess the effects of a Kegel exercise training program among women with urinary incontinence. The study was conducted at three government hospitals in Egypt’s Port Said city’s outpatient gynecological clinic. The intervention design was quasi-experimental. In total, 292 women with urine incontinence who visited the research sites made up the subjects. The necessary data were gathered using an interview questionnaire. Improvements in urinary incontinence and quality of life were positively correlated with daily Kegel exercise practice. Urinary incontinence has statistically significant positive correlations with age (*p* = 0.026), respiratory rate (*p* = 0.007), and body mass index (*p* = 0.026) as women grow older. Urinary incontinence, being single, and increasing pulse, however, had adversely significant negative correlations (*p* = 0.031 and 0.020, respectively). Urinary incontinence affects women’s overall wellbeing, particularly in the emotional and social spheres, as well as their quality of life and their ability to participate in normal everyday activities. Following the adoption of the Kegel exercise training program, there was a substantial improvement in both urine incontinence and quality of life.

## 1. Introduction

Millions of people around the world suffer with urinary incontinence, especially as adults [1,2]. The problem of unintentional urine loss (leakage) is known as urinary incontinence. Both genders can obtain the illness, but women are far more likely to do so. Stress incontinence, urge incontinence, and mixed incontinence are the different forms of UI.

The International Urogynecological Association and the International Continence Society have defined urge incontinence as the complaint of urine leakage associated with a sudden, intense, and difficult-to-resist urge to urinate, while stress incontinence is the complaint of urine leakage associated with coughing, sneezing, or physical exertion [3]. The combined symptoms of these two categories, known as mixed incontinence, are so widespread that they frequently coexist [4]. Stress urinary incontinence typically affects more than two thirds of patients with urine incontinence [5].

Pregnancy, vaginal delivery, pelvic surgery, obesity, and age are all common risk factors for urine incontinence in the general female population [2]. Research on the risk factors for urine incontinence in young nulliparous women, however, is scant [5]. It has been claimed that eating disorders, low body mass index, and few exercise hours may be plausible causes of UI [6].

Limitations on daily activities, sexual activity, and interpersonal relationships are caused by urinary incontinence [7]. The associated emotional issues, such as low self-esteem, depression, sadness, and embarrassment, eventually have a detrimental effect on quality of life [8,9,10]. Despite these unfavorable consequences, most women with urine incontinence do not seek medical attention because they believe it is a normal side effect of childbearing and aging rather than a major health issue [11].

Systematic reviews on pelvic floor muscle exercises have addressed all nonsurgical treatment options, including medication, for female urine incontinence, including urge, stress, and mixed forms [12,13]. Urinary incontinence is commonly treated with surgery, medication, behavioral therapies, and biofeedback. The treatment of choice for many patients with stress incontinence is behavioral therapy. Because it is non-invasive and risk-free, pelvic floor muscle training has been suggested as the primary treatment in many situations. Although behavioral therapy showed equal rates of improvement to medication, compliance rates were low [13,14].

Dr. Arnold Kegel created the Kegel exercise, a kind of pelvic floor muscle strengthening, in 1940. By contracting and relaxing the pubococcygeus, or pelvic floor muscle, during this exercise, the stress of urine incontinence was relieved. It is beneficial to enhance the strength of these muscles to contract and stretch as well as the muscle strength in the urethra [12,13].

Due to women being embarrassed to discuss this issue or believing the condition is incurable, urinary incontinence is still linked to low levels of women seeking care in medical facilities [15]. Additionally, numerous studies have offered explanations for why urinary incontinence treatment may be delayed, including the belief that the condition is an inevitable result of aging, childbirth, and/or stigma [16,17], psychosocial barriers (shame, embarrassment, fear of discrimination), fear of invasive treatment [18], and adequate self-coping mechanisms [19].

Between 20 to 60 percent of women in the Middle East reported having incontinence [20]. Only a few studies were conducted in Egypt. In a recent survey, 54.8% of women in the Assiut Governorate in Egypt reported having urine incontinence. It was 15%, 14.8%, and 25% more common to experience urge, stress, and mixed incontinence, respectively. However, UI consultations were infrequent (4%) [21].

In this study, women with urine incontinence were evaluated for their responses to a Kegel exercise training program. This study was the first to uncover the extent of urinary incontinence, types and severity of urinary incontinence, factors affecting urinary incontinence in women, and the impact of Kegel exercise on urinary incontinence in women visiting outpatient gynecological clinics in Port Said Governorate, Egypt.

## 2. Methods

### 2.1. Design

To investigate the impact of Kegel exercise on the treatment of women with urine incontinence, a quasi-experimental intervention study with pre–post assessment was conducted.

### 2.2. Setting

The study was conducted at three government hospitals in Port Said city’s outpatient gynecological clinic.

### 2.3. Subjects

Using convenience sampling, 292 women with urine incontinence were recruited for the study. 

Inclusion criteria: When a woman met the criteria for inclusion, she was asked to participate. The inclusion criteria were being a woman who (1) is between the ages of 17 and 45 and (2) can read Arabic. 

Exclusion criteria: Women were excluded (1) who were pregnant and (2) who had kidney or urinary infection within the previous four weeks, diabetes mellitus, genital prolapse, prior incontinence surgery, psychiatric disorders, mobility issues, genitourinary fistula, indwelling catheter, or inability to contract a pelvic muscle.

### 2.4. Instruments

The researchers created a structured interview questionnaire with two sections in order to gather the required data.

Section I comprises the following information about the women: (1) sociodemographic characteristics, such as age, marital status, level of education, and employment; (2) medical history, such as diabetes mellitus, hypertension, anemia, heart disease, and others, arthritis/rheumatism, hepatitis C, and prescription medications; and (3) surgical history, such as prolapse surgery, hysterectomy, appendectomy, thyroidectomy, oophorectomy, ovarian cyst, uterine fibroid, prolapse surgery, and removal of the back cartilage; (4) an obstetrical history, which covered gravidity, parity, the number of abortions, the number of live births, and the mode of delivery; (5) a physical examination, which looked at general appearance, vital signs, weight, and height.

Section II included Urinary Incontinence Scale. It was used to assess urinary incontinence patterns. The 34-item scale covers four domains. (1) Pattern Of Urinary Incontinence (5 items), (2) Stress Urinary Incontinence (9 items), (3) Urge Urinary Incontinence (6 items), and (4) Mixed Urinary Incontinence (14 items) as developed by Jayachandran [22]. Each item received a score of 0 for “never,” 1 for “rarely,” 2 for “sometimes,” and 3 for “often”. For the stress score, responses to things 1 through 9 are added up. For the urge score, responses to items 10 through 15 are added up. For the mixed urinary incontinence score, responses to items 16 through 30 are added up. If the percent score is 60% or more and less than 60%, the scales associated with each kind are regarded as common and uncommon, respectively. The Urinary Incontinence Scale was modified and translated into Arabic by the researchers.

Two sessions of the Kegel exercise training program were administered by the researchers to the subjects. It was delivered in a straightforward and succinct manner, utilizing several teaching techniques such as discussion and illustrations. The researchers gave the women a PowerPoint presentation, videos, and a role-playing exercise about successful Kegel exercises. The subjects carried out a demonstration back to the researchers to show that they understood and could perform the Kegel exercise.

A panel of seven specialists in the field of obstetrics and gynecological nursing evaluated the validity of the study instruments and the program’s contents, and revisions were made in light of their recommendations.

### 2.5. Pilot Study

In order to determine the feasibility of the study, the clarity of the data collection instrument, the dependability of study instruments, and the time required for data collection, a pilot study involving around 10% of the expected sample size (30 women) was conducted. The reliability of the tools was evaluated using the Cronbach’s alpha coefficient test (*r* = 0.85), which showed that each item had generally homogeneous components.

### 2.6. Fieldwork

Three phases were used to collect data from potential subjects who decided to participate in the study:

A. Assessment Phase: To collect data for two sections of the interview questionnaire, interviews were conducted. Between August 2019 and March 2020, data were collected in each facility over a two-day period each week. Each subject received an introduction from the researchers, who then verified her eligibility for the study and asked for her consent to participate. Every day, 3–6 subjects were questioned. About 20 to 30 min were allotted for each interview. The evaluation of subjects with urine incontinence was conducted using a pretest interview questionnaire. Additionally, this was used for the posttest eight weeks after the intervention.

B. Implementing Phase: The subjects were enrolled into the educational training program over the course of two sessions. Each session lasted between forty and fifty minutes. The researchers then evaluated, double-checked, and revised the clinical data of women.

C. Evaluation Phase: After implementing the treatment for 8 weeks, each individual with urine incontinence was assessed for her improvement by using an interview questionnaire to determine the degree of improvement.

### 2.7. Statistical Analysis

Variables from the interview questionnaire were entered and analyzed using the SPSS Statistics 28 software after data collection was completed (IBM Corp; Armonk, NY, USA, 2022). In contrast to continuous descriptive statistics, which were represented in terms of means, standard deviations, and minimum and maximum values, categorical variables were expressed as frequencies and percentages. Regression analyses with study variables were performed to examine factors that affect urinary incontinence. The body mass index was categorized as underweight: less than 18.5 kg/m^2^, normal: 18.5–24.9 kg/m^2^, overweight: 25.0–29.9 kg/m^2^, and obese: ≥30 kg/m^2^.

## 3. Results

The sociodemographic details of the participating women are shown in Table 1. They were 33.38 years old on average. In total, 48.6% of them (*n* = 142) were older than 25 to 35 years old, making up nearly half of the group. A total of 93.5 percent of the women who took part (*n* = 273) were married. Regarding education levels, 25.3% of the sample (*n* = 74) were illiterate, while 49.7% of the sample (*n* = 145) had completed secondary school. The majority of the women in the study (84.2%, *n* = 246) were housewives.

Table 2 also shows the surgical and obstetric histories of the women who were the subject of the study. Despite the fact that 25.7% (*n* = 75) of the participating women had hypertension, 4.1% (*n* = 12) had arthritis or rheumatism, and 2.1% (*n* = 6) had anemia, the bulk of the women (66.7%, *n* = 195) had no medical history. Hepatitis C and cardiovascular disease both had the lowest reported percentage of medical conditions at 0.7% (*n* = 2).

Nearly two-thirds (74.7%, *n* = 218) of subjects reported having no surgical histories. The appendectomy rate was 10.9% (*n* = 32), the cholecystectomy rate was 6.1% (*n* = 18), and the hysterectomy rate was 2.7% (*n* = 8). The lowest recorded percentages (2.1%, 1.4%, 0.7%, 0.7%, and 0.7%, respectively) were associated with undergoing ovarian cyst removal, thyroidectomy, oophorectomy, uterine fibroid removal, and back cartilage discectomy. 

In total, 143 out of the 292 women who participated in the study, or 49%, said they had given birth three or more times. There were zero to nine previous pregnancies in total. In a similar vein, 46.2% (*n* = 135) of women who gave birth to children after 20 weeks of gestation had performed it three or four times. Regarding the assessment of their abortion history, nearly three-fifths of them (59.6%, *n* = 174) stated that they had never had an abortion. In total, 46.2% (*n* = 135) of the population had one to two living children, according to Table 2. One hundred and eighty people (*n* = 61.6%; method of delivery: cesarean) reported having cesarean births.

The findings of the physical examination among the participating women are listed in Table 3. A normal pulse rate (between 60 and 100 beats per minute) was present in the majority of them (96.6%, *n* = 282), while a moderate percentage (77.7%, 90.8%, and 99.3%, respectively) had normal blood pressure, respiration, and temperature. In terms of weight, 68.8% (*n* = 201) had a weight that fell between 60 and 90 kg, whereas 48.6% (*n* = 142) had a height that fell between 160 and 170 cm.

**Figure 1:**
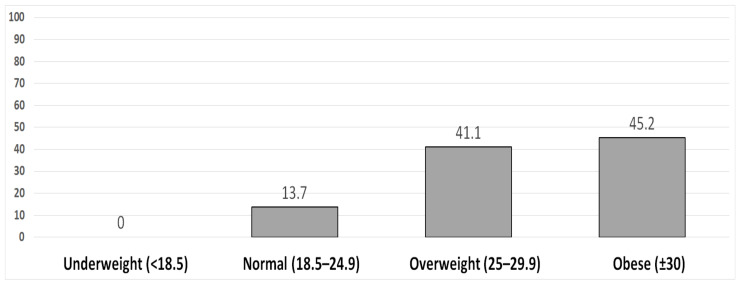
Subjects’ body mass index (kg/m^2^).

**Figure 2:**
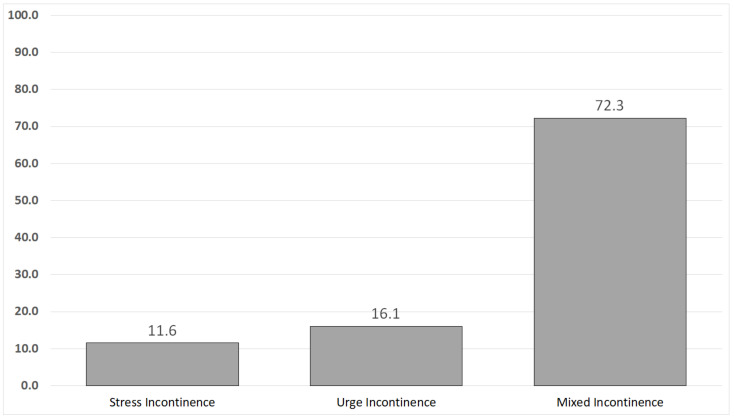
The type of urinary incontinence reported by subjects.

No subjects were found to be underweight, according to Figure 1’s body mass indices, while 41.1% (*n* = 120) and 45.2% (*n* = 132) were determined to be overweight and obese, respectively. Nearly two-thirds of the women (72.3%, *n* = 211) had mixed urine incontinence, 11.6% (*n* = 34) had stress incontinence only, and 16.1% (*n* = 47) had urge incontinence solely Figure 2.

The trend of urine incontinence and quality of life among the investigated women during the course of the program phases is shown in Table 4. Improvements in urine leakage rate and amount were shown to be statistically significant (*t* = 1.00, *p* < 0.001) after a Kegel exercise training program (i.e., posttest) compared to pretest scores. After completing the Kegel exercise training program, the average felt quality-of-life score (out of 10) was much higher (8.2) than it was before the test (6.67).

Results of the regression test for variables influencing female urine incontinence are shown in Table 5. Urinary incontinence and age (*t* = 1.169, *p* = 0.026), respiratory rate (*t* = 2.733, *p* = 0.007), and body mass index (*t* = 2.243, *p* = 0.026) all showed statistically significant positive relationships. However, there were significant negative correlations between urine incontinence, being single, and increasing pulse (*p* = 0.031 and 0.020, respectively).

## 4. Discussion

With a mean age of 33.38 (and standard deviation of 6.9), our research showed that almost half of the women we studied were older than 25 to 35. Our findings are consistent with the research by Joshi et al. [23] that examined the effectiveness of postpartum Kegel exercises in preventing and treating stress incontinence in postpartum women. They discovered that the majority of the participating women were between the ages of 18 and 29. On the other hand, according to other studies [24,25], subjects aged 45 and older were more likely to experience stress incontinence. The inconsistent results could be a result of the parity level heterogeneity among the various study samples. Therefore, regardless of their age group, it is extremely important for women to perform Kegel exercises to strengthen the pelvic muscle and reduce the chance of urine incontinence [26].

The majority of the participating women were married and housewives, according to the study. Nearly half of them had completed secondary school. These results shadow the findings of Mohamed et al. [25] who reported that the majority of the participant women were married and nearly half of them had secondary education. From the perspective of the researchers, this is expected because many women in Egypt tend to quit their education at the secondary school level and marry young.

Regarding the surgical history, the present finding revealed that the majority of women had no surgical history. This conclusion is consistent with Navarro-Brazález et al.’s [27] finding that only 8.5% of participant women had undergone prior pelvic floor procedures, indicating that the majority had no surgical background. Healthcare professionals may be more likely to assess for urine incontinence regardless of a patient’s history of pelvic floor surgery if they are aware that urinary incontinence is not always linked to prior pelvic procedures.

In terms of the number of pregnancies, the most current study showed that nearly half of the participating women had three to four pregnancies, and nearly half had delivered three to four times. These findings are in agreement with those of Mohamed et al. [25]. They noted that patients who were pregnant and gave birth more than three times were statistically more likely to experience stress incontinence. Our study also revealed that more than half of the participating women had never had an abortion before. This result is consistent with that of Elbana et al. [24], who found that more than 75% of subjects had never had an abortion.

More than two thirds of the recruited subjects for the current study’s analysis of women’s weight had weights between 60 and 90 kg, and nearly half had heights between 160 and 170 cm. These results agree with those of Zlü et al. [28]. In the same vein, the most percentages of the studied women were obese, with a body mass index of more than 30 kg/cm^2^, according to the current study. Fitz et al. [29] found that the majority of the women they surveyed were close to being obese, with a mean BMI of around 29. An increasing body mass index should be taken into account when determining whether to test for urine incontinence. 

The current study’s findings on urine incontinence type showed that roughly three-quarters of the investigated women had mixed urinary incontinence. This result agrees with the findings of the Abd El-Aty and Hassan study [26]. More than half of the women in the study had a mixed kind of urine incontinence, the researchers noted.

Results of this study demonstrated that after (post) a Kegel exercise training program compared to the pretest phase, there were statistically significant improvements in the pattern of urine incontinence among the tested women. Kegel exercises are one of the most important methods for rehabilitating and bolstering pelvic floor muscles and facilitating urine storage [26]. After three months of the therapy, they reported that more than one-third of the women had been fully cured and two-thirds had improved. Similar results were found in the Mohamed et al. [25] study, which showed that there was a highly statistically significant difference between the control and study groups in terms of the mean strength of the pelvic floor muscles, the symptoms of urinary incontinence, and the effect on quality of life.

The quality of life also improved after the training program was fully implemented. After implementing pelvic floor muscle training, Radzimiska et al. [30] noticed a statistically significant increase in the quality of life in the majority of the studied women in the experimental groups. According to Soliman et al. [31], urine incontinence significantly lowers the quality of life for women. According to researchers, having urine incontinence puts restrictions on going to public places, taking leisure trips, and even going to work because it disrupts social life. Additionally, in Egypt, women who have such complaints never discuss them with anybody, not even their spouse, out of shame at having a urine scent or being wet in public. As a result, Kegel exercises, which strengthen the muscles in the pelvic floor and enhance muscle tone, are frequently used as the first non-pharmacological treatment option [32,33].

Regarding the variables influencing the study of women’s urine incontinence, urinary incontinence was statistically significantly correlated with age, marital status, pulse, respiration, and body mass index. This result is consistent with those of studies by Concepcion et al. [34] and Mohamed et al. [25], which found a strong relationship between aging and abnormal BMI and a higher likelihood of women reporting urine leakage. In addition, a systematic review and meta-analysis conducted by Batmani et al. [2] found that women’s age, obesity, and hypertension are the most significant factors impacting the occurrence of urine incontinence. Additionally, among the studied women, Soliman et al. [31] discovered a statistically significant relationship between urine incontinence and age, education levels, and body mass index. It is important to highlight that a high BMI may be caused by edema rather than excessive body fat. Additionally, because of the aging process, there is a statistically significant positive link between urine incontinence and age.

The subjects’ core stability should be addressed by motivating them to engage in workouts that increase their lumbar–pelvic–hip muscle complex’s capacity to control lower trunk movement and preserve vertebral column stability. To further analyze the development of musculo-fascial deficits in women with pelvic floor dysfunction after vaginal delivery, healthcare providers may suggest employing magnetic resonance imaging, which offers a thorough display of the pelvic floor structures responsible for appropriate pelvic floor architecture.

Assessment of pelvic floor contractions can be performed using real-time transabdominal ultrasonography. It is a valid, reliable, and non-invasive method for analyzing pelvic structural movement during contraction. While the ability to contract and endurance of muscles may be precisely examined, the power of muscular contractions cannot. The pelvic floor muscles can also be assessed with surface electromyography. Additionally, as reliable instruments for determining the strength of a pelvic floor muscle contraction, manometry and dynamometry may be preferable to digital palpation [35].

The findings of this study can be used by additional researchers who wish to include additional research sites and conduct longer Kegel exercise training programs. For all women who experience urine incontinence, applying pelvic muscle training is strongly advised as a standard intervention across all outpatient gynecological clinics to reduce its incidence.

The nurses in Egypt should be interested in incorporating a Kegel exercise training program that targets the pelvic muscle for all women with urinary incontinence in gynecological clinics. The findings of this study are foundational for future studies. 

To encourage early treatment of urine incontinence among women, it is strongly advised that health education programs be implemented in health facilities. These programs should also inform women about the value of Kegel exercises and how to conduct them. Another option is to distribute pamphlets on preventing urine incontinence in gynecological clinics and healthcare facilities.

## 5. Conclusions

Women of all ages experience the uncomfortable and common condition known as urinary incontinence. Although urine incontinence is not a fatal condition, it has an impact on women’s overall health, particularly in the emotional and social spheres. The quality of life was also significantly impacted, and daily activities were constrained. This study looked into how the Kegel exercise training program affected urine incontinence in female subjects. We noticed that the Kegel exercise training program had improved the symptoms of female urine incontinence. Additionally, there were statistically significant negative correlations between urine incontinence and being single and elevated pulse rate, as well as statistically significant positive relationships with age, respiration rate, and body mass index.

### Strengths and Limitations

The study’s advantages include a large sample size and a diverse geographic dispersion of the patients who were enrolled in one of Egypt’s largest cities. According to our knowledge, this is the first study to have studied and highlighted various major findings concerning urinary incontinence in Port Said. Convenience sampling is one of the limitations of this study, and it can only give a picture of the situation in our sample. The history and testing threats may also have an impact on the validity of posttest responses.

## Figures and Tables

**Table 1:** Distribution of the subjects according to their sociodemographic characteristics.

	No.	%	Min	Max	Mean	SD
Age (Years)			17	45	33.38	6.9
17–25	42	14.4%				
>25–35	142	48.6%				
>35–45	108	37.0%				
Level of Education						
Illiterate	74	25.3%				
Reading and Writing	16	5.5%				
Primary Education	22	7.5%				
Secondary Education	145	49.7%				
High Education and Above	35	12.0%				
Marital Status						
Single	10	3.4%				
Married	273	93.5%				
Widowed	4	1.4%				
Divorced	5	1.7%				
Employment Status						
Housewives	246	84.2%				
Working	46	15.8%				

**Table 2:** Distribution of the subjects according to their medical, surgical, and obstetric histories.

	No.	%	Min	Max	Mean	SD
Medical History						
None	195	66.7%				
Hypertension	75	25.7%				
Cardiac Disease	2	0.7%				
Arthritis/Rheumatism	12	4.1%				
Anemia	6	2.1%				
Hepatitis C	2	0.7%				
Surgical History						
None	218	74.7%				
Hysterectomy	8	2.7%				
Appendectomy	32	10.9%				
Cholecystectomy	18	6.1%				
Thyroidectomy	4	1.4%				
Oophorectomy	2	0.7%				
Ovarian Cyst Removal	6	2.1%				
Uterine Fibroid Removal	2	0.7%				
Back Cartilage Discectomy	2	0.7%				
No. of Gravida			0	9	3.22	1.67
None	21	7.2%				
1–2	72	24.7%				
3–4	143	49.0%				
≥5	56	19.1%				
No. of Parity			0	9	2.61	1.4
None	25	8.6%				
1–2	112	38.4%				
3–4	135	46.2%				
≥5	20	6.8%				
No. of Abortions			0	4	0.62	0.93
0	174	59.6%				
1	78	26.7%				
2	24	8.2%				
3	10	3.4%				
4	6	2.1%				
No. of Living Children			0	11	2.72	1.58
1–2	135	46.2%				
3–4	131	44.9%				
5–6	20	6.8%				
≥6	6	2.1%				
Mode of Delivery						
None	25	8.6%				
Cesarean	180	61.6%				
Vaginal	87	29.8%				

**Table 3:** Distribution of the subjects according to their physical examination.

	No.	%	Min	Max	Mean	SD
Pulse						
<60	8	2.7%				
60–100	282	96.6%				
>100	2	0.7%				
Blood Pressure						
Below Normal	19	6.5%				
Normal	227	77.7%				
Above Normal	46	15.8%				
Respiration Rate						
<12						
12–20	265	90.8%				
>20	27	9.2%				
Temperature						
<36.5	2	0.7%				
36.5–37.5	290	99.3%				
>37.5	0	0.0%				
Weight			55	138	84.77	14.54
<60 kg	4	1.4%				
60–90 kg	201	68.8%				
>90 kg	87	29.8%				
Height			150	192	164.97	8.94
<160	81	27.7%				
160–170	142	48.6%				
>170	69	23.6%				

**Table 4:** Distribution of the subjects according to their pattern of urinary incontinence and quality of life throughout the program phases.

	Pretest		Posttest		*t*	Sig.
	No.	%	No.	%		
Rate of Urine Leakage					1.000	0.000
Never	0	0.0%	0	0.0%		
About once a week or less often	46	15.8%	53	18%		
Two or three times a week	62	21.2%	86	30%		
About once a day	24	8.2%	36	12%		
Several times a day	145	49.7%	104	36%		
All the time	15	5.1%	13	4%		
Amount of Urine Leakage					1.000	0.000
None	0	0.0%	0	0.0%		
A small amount	218	74.7%	224	77%		
A moderate amount	50	17.1%	46	16%		
A large amount	24	8.2%	22	7%		
Quality of Life					1.000	0.000
<4	18	6%	11	3.8%		
4–6	58	20%	52	17.8%		
>6	216	74%	229	78.4%		
Min–Max	0–10		0–10			
Mean ± SD	6.67 ± 1.72		8.20 ± 2.16			

**Table 5:** Regression test for factors affecting urinary incontinence *.

	Unstandardized Coefficients		Standardized Coefficients	*t*	Sig.
	B	Std. Error	Beta		
(Constant)	−6.134	6.078			
Age	0.008	0.007	0.081	1.169	0.026 *
Education	−0.042	0.030	−0.087	−1.407	0.161
Marital status	−0.171	0.079	−0.133	−2.163	0.031 *
Employment Status	−0.138	0.115	−0.073	−1.205	0.229
No. of Para	−0.107	0.057	−0.218	−1.867	0.063
No. of Abortion	−0.033	0.044	−0.044	−0.741	0.459
No. of Children	0.017	0.048	0.039	0.351	0.726
Mode of Delivery	−0.017	0.076	−0.014	−0.217	0.828
Pulse	−0.019	0.008	−0.264	−2.344	0.020 *
BP	0.093	0.080	0.079	1.165	0.245
Respiratory	0.090	0.033	0.269	2.733	0.007 *
Temperature	0.236	0.174	0.119	1.357	0.176
BMI	0.202	0.090	0.193	2.243	0.026 *

* Statistically significant at *p* < 0.05.

## Data Availability

The data presented in this study are available on request from the corresponding author. The data are not publicly available due to privacy restrictions.
